# Low Amplitude Boom-and-Bust Cycles Define the Septoria Nodorum Blotch Interaction

**DOI:** 10.3389/fpls.2019.01785

**Published:** 2020-01-31

**Authors:** Huyen T. T. Phan, Darcy A. B. Jones, Kasia Rybak, Kejal N. Dodhia, Francisco J. Lopez-Ruiz, Romain Valade, Lilian Gout, Marc-Henri Lebrun, Patrick C. Brunner, Richard P. Oliver, Kar-Chun Tan

**Affiliations:** ^1^ Centre for Crop and Disease Management, School of Molecular and Life Sciences, Curtin University, Perth, WA, Australia; ^2^ ARVALIS Institut du Végétal Avenue Lucien Brétignières, Bâtiment INRA Bioger, Thiverval-Grignon, France; ^3^ UMR INRA Bioger Agro-ParisTech, Thiverval-Grignon, France; ^4^ Plant Pathology, Institute of Integrative Biology, ETH Zurich, Zurich, Switzerland

**Keywords:** septoria nodorum blotch, SSR, effector, population, wheat

## Abstract

**Introduction:**

Septoria nodorum blotch (SNB) is a complex fungal disease of wheat caused by the Dothideomycete fungal pathogen *Parastagonospora nodorum*. The fungus infects through the use of necrotrophic effectors (NEs) that cause necrosis on hosts carrying matching dominant susceptibility genes. The Western Australia (WA) wheatbelt is a SNB “hot spot” and experiences significant under favorable conditions. Consequently, SNB has been a major target for breeders in WA for many years.

**Materials and Methods:**

In this study, we assembled a panel of 155 WA *P. nodorum* isolates collected over a 44-year period and compared them to 23 isolates from France and the USA using 28 SSR loci.

**Results:**

The WA *P. nodorum* population was clustered into five groups with contrasting properties. 80% of the studied isolates were assigned to two core groups found throughout the collection location and time. The other three non-core groups that encompassed transient and emergent populations were found in restricted locations and time. Changes in group genotypes occurred during periods that coincided with the mass adoption of a single or a small group of widely planted wheat cultivars. When introduced, these cultivars had high scores for SNB resistance. However, the field resistance of these new cultivars often declined over subsequent seasons prompting their replacement with new, more resistant varieties. Pathogenicity assays showed that newly emerged isolates non-core are more pathogenic than old isolates. It is likely that the non-core groups were repeatedly selected for increased virulence on the contemporary popular cultivars.

**Discussion:**

The low level of genetic diversity within the non-core groups, difference in virulence, low abundance, and restriction to limited locations suggest that these populations more vulnerable to a population crash when the cultivar was replaced by one that was genetically different and more resistant. We characterize the observed pattern as a low-amplitude boom-and-bust cycle in contrast with the classical high amplitude boom-and-bust cycles seen for biotrophic pathogens where the contrast between resistance and susceptibility is typically much greater. Implications of the results are discussed relating to breeding strategies for more sustainable SNB resistance and more generally for pathogens with NEs.

## Introduction

*Parastagonospora nodorum* causes septoria nodorum blotch (SNB) of wheat (*Triticum* spp.) (Solomon et al., 2006). The fungus causes significant yield losses in many wheat growing regions world-wide (Eyal et al., 1987; Loughman, 1989; Oliver et al., 2009). *P. nodorum* reproduces sexually before the start of the growing season forming air-borne ascospores that can disperse over long distances and asexually throughout the growing season for short distance rain-splash dispersal (Eyal et al., 1987). Mixed reproduction allows the rapid production of adapted recombinant genotypes *via* sexual reproduction and their fixation and multiplication in the asexual phases (Brasier et al., 1999; McDonald and Linde, 2002).

The development of SNB is dictated by interactions between several proteinaceous necrotrophic effectors (NEs) secreted by *P. nodorum* and dominant susceptibility genes in the host (Friesen and Faris, 2010; Oliver et al., 2012). A compatible interaction results in host tissue death and disease. Three NE genes have been identified and fully characterised for their role in SNB. *ToxA* encode a protein that causes necrosis on wheat varieties that carry the dominant susceptibility gene *Tsn1* (Friesen et al., 2006). Two other wheat pathogens also contain *ToxA*; *Pyrenophora tritici-repentis* (*Ptr*) (Ciuffetti et al., 1997) and *Bipolaris sorokiniana* (*Bs*) (McDonald et al., 2018). A global survey of *P. nodorum* isolates identified 17 *SnToxA* haplotypes encoding nine unique protein isoforms (McDonald et al., 2013). *Tox1* encodes a cysteine-rich protein with a chitin binding-like motif at the carboxyl terminus. Tox1–induced chlorosis requires the expression of the host sensitivity gene *Snn1* located on wheat chromosome 1BS (Liu et al., 2004; Liu et al., 2012). *Tox3* encodes a small cysteine-rich protein and sensitivity is controlled by *Snn3* which is located on wheat chromosome 5BS (Shi et al., 2016). These effectors possess multiple protein isoforms (McDonald et al., 2013).

SNB remains a problematic disease worldwide despite a greater understanding on the role of NEs in the establishment of disease in the *P. nodorum* — wheat pathosystem. In Australia, host resistance in current cultivars is partial at best (Zaicou-Kunesch et al., 2018). The degree of resistance is not a simple reflection of the number of matching NEs in the fungus and susceptibility genes in host cultivars. Molecular genetic analyses have shown that SNB is impacted by the complex interactions of multiple wheat QTLs (Shankar et al., 2008; Friesen and Faris, 2010; Tan and Oliver, 2017). SNB is further complicated by differences in effector isoform activity. For *ToxA* at least, protein isoforms exhibit large differences in necrosis-inducing activities (Tan et al., 2012). Isoforms that induced the most rapid necrosis on *Tsn1* wheat caused the most fungal sporulation.

The population structure of the Western Australia (WA) *P. nodorum* population has been examined in several studies using various genetic markers. Murphy et al. (2000) analyzed isolates collected over 13 years using four restriction-fragment-length-polymorphism probes and observed a high level of genetic diversity but no distinct subpopulations. Stukenbrock et al. (2006) examined the global migration pattern of the pathogen using EST-SSR markers (Stukenbrock et al., 2005). A high global migration rate of *P. nodorum* was observed, with the Australian population acting as a sink for foreign immigrants. Like Murphy et al. (2000); Stukenbrock et al. (2006) also observed a high level of genotypic diversity within the Australian *P. nodorum* population even though it was sampled from a single field in 2001.

The aim of this study was to undertake a more complete analysis of the genetic structure of the population using a set of isolates collected over a 44-year period. Such a collection collected over a long-time span is extremely rare and mainly restricted to well-studied model fungi like *Puccinia triticina* (Ordonez and Kolmer, 2009) and *Neurospora crassa* (Turner et al., 2001). Another objective was to investigate differences or trends in virulence of the structured populations and seek for their explanations. By combining pathogen population genetic data with data on the cultivation of wheat, we hoped to shed light on the microevolutionary process operating in time and space.

## Materials and Methods

### Fungal Reisolation

**Figure 1:**
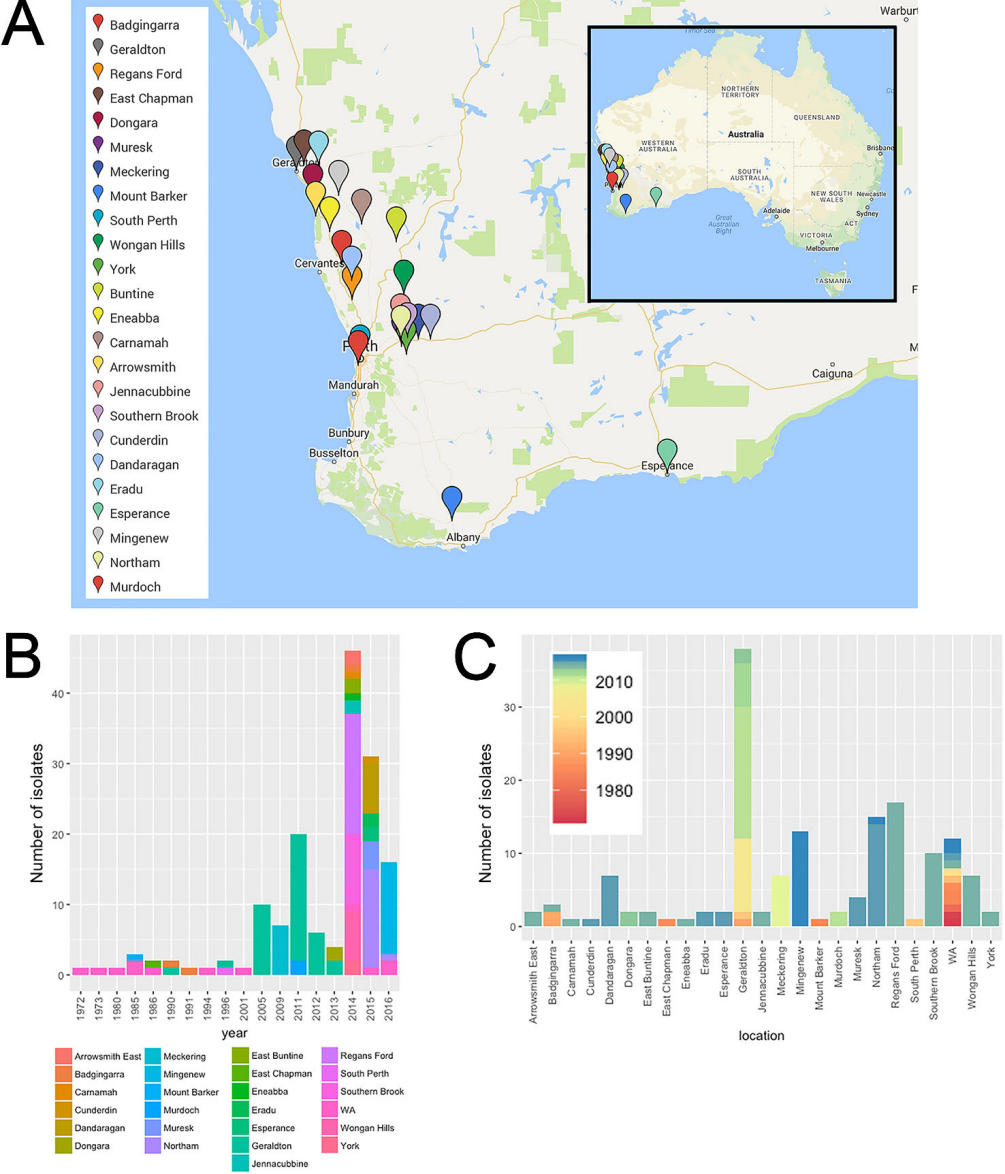
Spatial-longitudinal distribution of Australian *P. nodorum* isolates collected between 1972-2016: **(A)** Sampling sites (Google Maps, CA, USA) isolates with unknown collection location in WA were designated as “WA” and were not included in the map; **(B)** number of isolates per sampling year; **(C)** per sampling location.

A set of 155 Australian *P. nodorum* isolates collected in WA between 1972 and 2016 was used in this study (**Table S1**, **Figure 1**). Fifty-five were obtained from the Department of Primary Industries and Regional Development (DPIRD). A further 100 were isolated from leaf samples with SNB symptoms using two following methods: the traditional method obtained 73 single pycnidial isolations as previously described (McDonald et al., 1994); and the second method used 10 µg/ml boscalid to suppress *Ptr*. To do this, the infected leaf was embedded onto tap water agar supplemented with 10 mg/L boscalid plus 100 mg/L ampicillin, 30 mg/L streptomycin, 50 mg/L neomycin sulphate to suppress bacterial growth. Growing hyphae were then transferred onto V8PDA agar medium supplemented with the same antibiotics and grown until pycnidiation to generate pure cultures. To test whether this isolation method biased towards fungicide tolerant isolates, the EC_50_ concentration of boscalid was determined using a microtitre plate assay (Mair et al., 2016).

Eighteen *P. nodorum* isolates from France isolated as previously described (McDonald et al., 1994) one from Denmark and four from the USA were also included in the study. One *Ptr*, one *Parastagonospora avenae* f.sp. *triticae* (*Pat*), one *Phoma* sp. (15FG039), two *Pyrenophora teres* f. sp. *maculata* (*Ptm*), and one *Pyrenophora teres* f. sp. *teres* (*Ptt*) isolates were included in the study as phylogenetically distinct outgroup controls.

### SSR Marker Design

Twelve SSR markers designed from *P. nodorum* ESTs were tested (Stukenbrock et al., 2005). Only SNOD1, SNOD23, SNOD3, SNOD5, and SNOD8 demonstrated consistent amplification and were subsequently used in this study. We designed 25 SSR primer pairs (**Table S2**) from the 20 largest genomic scaffolds of the *P. nodorum* SN15 assembly to increase genome-wide marker coverage (Syme et al., 2016).

Nei's diversity index (H_exp_) was used to test for the effect of each marker used in this study (Nei, 1978). The genetic diversity of the *P. nodorum* collection was calculated omitting each of the markers in turn to assess the effect of each SSR marker. H_exp_ indicated that none of these markers gave biased genetic diversity values as they were in a similar range and compatible to that derived from the four EST-SSR previously published markers (SNOD1, SNOD3, SNOD5, and SNOD8).

### SSR Genotyping

SSR genotypes were obtained using a multiplex-ready PCR technique (Hayden et al., 2008). PCR products were subsequently electrophoresed using an ABI-PRISM 3730xl automated sequencer (Applied Biosystems, CA, USA). PCR amplicon lengths were determined by GeneMarker V1.91.

### Population Analyses

Amplicon lengths representing SSR alleles were rounded to the nearest multiple of the repeat length and analysed using the R statistical programming language (Development Core Team R, 2008). To assess the number of loci required to discriminate individuals in the population, genotype accumulation curves were calculated using poppr v2.6.0 (Kamvar et al., 2014). Bootstrapped (*n* = 100) phylogenetic trees were estimated using Bruvo's distance (Bruvo et al., 2004) and unweighted-pair-group-method-with-arithmetic-mean (UPGMA) hierarchical clustering algorithm in poppr. Trees were rooted using four *Pyrenophora* sp., *P. avenae* and a *Phoma* sp. as outgroups.

Summary statistics including: allele frequency, Simpson's index (λ), H_exp_, and evenness for SSR loci were obtained using poppr (Grunwald et al., 2003). Uninformative markers (i.e., those with < 2 variant isolates or where 99% of isolates possess one major allele) were identified using poppr and removed from further analysis.

SSR variation between isolates was summarised using principal component analysis (PCA) from the ade4 and adegenet R packages (Chessel et al., 2004). Isolates were clustered based on SSR data using snapclust (Jombart et al., 2010).

Relationships between the clustered groups of isolates identified by snapclust and the alleles contributing to their separation were dissected using discriminant analysis of principal components (DAPC) (Jombart et al., 2010) in adegenet for the Australian population. Given a set of isolates with assigned groups, DAPC finds linear functions of PCs of the SSR genotype data that maximise differences between groups and minimise differences within groups. The number of PCs to include in the model was determined by performing cross-validation DAPC using a range of PC retention numbers each cross-validated 50 times (90%:10% training: validation stratified splits) so that it minimised the mean squared error of reclassification. Alleles with loading values above 0.03 were considered to have a major influence in discriminating isolate groups. Posterior probabilities of group membership resultant from DAPC models were used to assess groupings, detect possible admixture between groups and identify anomalous isolates.

For each identified population, the number of multilocus genotypes (MLG) observed, number of expected MLG, Shannon-Wiener Index of MLG diversity (Shannon, 1997), Stoddart and Taylor's index of MLG diversity (Stoddart and Taylor, 1988), λ index (Simpson, 1949) and H_exp_ (Nei, 1978) were calculated using poppr (Kamvar et al., 2014). These parameters were calculated taking into account differences in sample sizes between these groups. A clone-corrected dataset was generated by retaining a representation of duplicated isolates identical in SSR genotypes. The Prevosti's distance model-free method was used to calculate the genetic variation between groups observed from the Australian *P. nodorum* panel (Prevosti et al., 1975).

### Determination of the Mating Genotype and Index-of-Association (I_A_) in *P. nodorum* Isolates

Mating types in *P. nodorum* isolates were determined by PCR amplification of genomic DNA with specific primers MAT1-1/2 and MAT2-1/2 (Bennett et al., 2003). A Chi square test (χ^2^) was used to determine if the observed *MAT1*:*MAT2* ratio departed from the standard 1:1. I_A_, rbarD (*ȓ_d_*) and I_A_ test which uses 999 permutation of the data were deployed to determine if populations are in linkage disequilibrium (Smith et al., 1993) using poppr (Kamvar et al., 2014).

### Commercial Wheat Varieties and Disease Rating

SNB resistance ratings of wheat cultivars rated from 1990 until 2018 were obtained from the DPIRD historical archive as ‘Crop Variety Guides’ (https://www.agric.wa.gov.au/library) and was converted to a numerical system for calculations: 10, very susceptible; 9, susceptible-very susceptible; 8, susceptible; 7, moderately susceptible-susceptible; 6, moderately susceptible; 5, moderately resistant-moderately susceptible/intermediate.

The *P. nodorum* collection period into three eras corresponding to major shifts in wheat cultivar adoption. Crop planting data was obtained from DPIRD as “Crop Variety Guides” (https://www.agric.wa.gov.au) based on CBH Group reports (https://www.cbh.com.au). Coefficient correlation was determined independently for each period based on the *P* values of the test between frequency of each groups and frequency for all top-grown wheat lines for Era I (1984–2001), II (2001–2013) and III (2013–present).

### Whole Plant Seedling Infection Assay

Three isolates from each group (Group 1: FG63, MuS3, FG49; Group 2: Meck1, WAC13077, WAC13955; Group 3: WAC8635, WAC13418, SN15; Group 4: WAC13404, WAC13632, WAC13525; Group 5: 16FG165, 16FG167, 16FG168) were chosen for seedling infection assay on seven top-grown wheat lines from the three eras (Era I: Halberd, Eradu, Cadoux; Era II: Carnamah, Calingiri, Wyalkatchem; Era III: Mace). The disease assay for seedling plants was carried out following the method described in Solomon et al. (2003). Briefly pycnidiospore suspension was prepared to a concentration 1x10^6^ spores/ml in 0.05% (w/v) gelatine. All wheat lines were planted in a randomised design in three replicates. Two-week-old seedlings were sprayed with the spore-suspensions until runoff and kept under 100% relative humidity at 21°C under a 12-h photoperiod for 48 h. The plants were kept moist using a fine misting system in the growth chamber for five more days before disease symptom was scored in a scale from 1 to 9. A score of one indicates no disease symptoms whereas a score of nine indicates a fully necrotised plant. Significant differences in disease scores for each group in each era and their interactions were determined based on analysis of variance (ANOVA) and Tukey's Post Hoc test using statistical functions in core R and agricolae v1.3 R package (https://cran.r-project.org/web/packages/agricolae). Each isolate was inoculated on the individual cultivars with three replicates and then the data put together for variance analysis between eras.

## Results

### Isolation and Assembly of the *P. nodorum* Isolate Panel

A collection of 155 WA (this study; Solomon et al., 2004b; Syme et al., 2018) and 23 non-Australian [this study; (Friesen et al., 2006; Syme et al., 2018; Richards et al., 2019)] *P. nodorum* isolates, plus single isolates of five closely-related species were used in this study (**Figure 1**). WA isolates were collected from 24 known locations across the WA wheat belt. Most isolates were collected from 2005 onwards comprising of 93.6% of the entire WA collection. Isolates from Geraldton, collected between 1990 and 2013, is the largest regional group which comprised of 24.5% of isolates in the WA collection. The 12 WA isolates collected between 1972 and 2016 do not have sampling site information and were assigned as “unknown WA” (**Figure 1**; **Table S1**). Through the sampling process, we developed a new method to isolate *P. nodorum* from fresh infected leaf tissues using 10 µg/ml boscalid (**Figure S1**). This method permitted the separation of co-infecting *Ptr* (**Figure S1**, **Table S3**).

### SSR Marker Development

We developed 25 new microsatellite loci from the genome sequence of the *P. nodorum* reference isolate SN15 and added 5 EST_SSR markers from Stukenbrock et al. (2005). A genotype-accumulation-curve indicated that 27 loci developed in this study and from Stukenbrock et al. (2005) was sufficient to capture all observed genetic diversity existing in the Australian *P. nodorum* collection (**Figure S2A**). Two SSR markers (SSR3 and SNOD23) were found to be uninformative within *P. nodorum* isolates and were omitted from further analysis. The genetic diversity of the *P. nodorum* collection was calculated omitting each of the markers in turn to assess the effect of each SSR marker. H_exp_ indicated that none of these markers gave biased genetic diversity values (**Figure S2B**) as they were in a similar range and compatible to those derived from the four previously published EST-SSR markers (SNOD1, SNOD3, SNOD5, and SNOD8).

A clonality test of the Australian *P. nodorum* isolates revealed two pairs with identical SSR profiles (1. 16FG161 and 16FG162; 2. 16FG169 and 16FG170). Isolates 16FG162 and 16FG170 were removed, thus retaining 153 non-clonal isolates for further analyses.

The properties of the 28 informative SSR markers were calculated from the clone-corrected panel (**Table 1**). An average of 14.7 alleles/locus were observed. Four loci (SSR14, SSR16, SSR21 and SSR27) had a high λ index (> 0.90). Overall genetic diversity with an average H_exp_ of 0.71 was reported (**Table 2**).

**Table 1:** Number of alleles detected, λ index, H_exp_, and evenness for each SSR marker.

Marker	Allele number	λ	H_exp_	Evenness
SNOD1	13	0.28	0.28	0.33
SNOD23	4	0.04	0.04	0.32
SNOD3	5	0.47	0.47	0.80
SNOD5	10	0.64	0.64	0.67
SNOD8	10	0.33	0.34	0.45
SSR1	11	0.82	0.83	0.82
SSR3	1	0.00	0.00	NA
SSR4	22	0.85	0.85	0.57
SSR5	9	0.77	0.77	0.74
SSR6	7	0.33	0.33	0.53
SSR7	20	0.87	0.88	0.66
SSR8	8	0.73	0.74	0.72
SSR9	19	0.78	0.78	0.60
SSR10	16	0.86	0.86	0.74
SSR12	12	0.77	0.78	0.68
SSR14	33	0.91	0.92	0.61
SSR15	14	0.87	0.88	0.78
SSR16	26	0.93	0.94	0.82
SSR17	18	0.77	0.77	0.52
SSR18	11	0.72	0.72	0.64
SSR19	16	0.51	0.51	0.44
SSR20	6	0.63	0.64	0.82
SSR21	27	0.92	0.93	0.72
SSR22	13	0.78	0.79	0.59
SSR23	14	0.87	0.88	0.79
SSR24	14	0.66	0.66	0.52
SSR25	11	0.68	0.68	0.54
SSR26	10	0.29	0.29	0.35
SSR27	22	0.90	0.91	0.70
SSR28	15	0.77	0.78	0.61
Mean	13.90	0.66	0.66	0.62

**Table 2:** Gene, genotypic diversity and linkage disequilibrium of Australian *P. nodorum* isolates and its associated discriminant analysis of principal components (DAPC) groups.

Group	*n*	Ĝ	λ	H_exp_
All	153	98.71	0.99	0.71
Group 1	66	100.00	0.98	0.67
Group 2	56	100.00	0.98	0.71
Group 3	7	100.00	0.86	0.31
Group 4	14	87.50	0.92	0.20
Group 5	10	100.00	0.90	0.09

**Figure 2:**
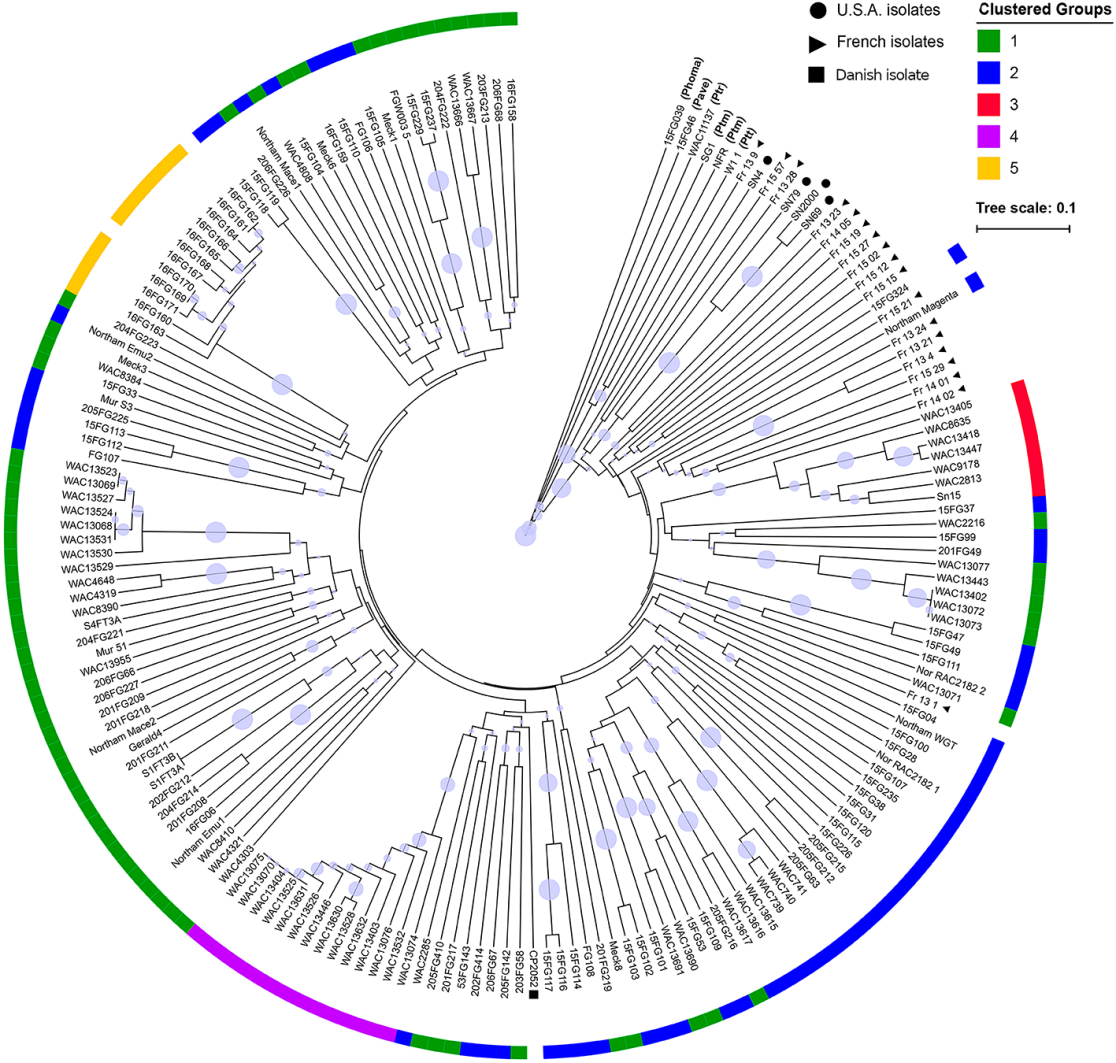
A UPGMA tree of 184 P*. nodorum* and other species constructed using Bruvo's distance with non-parametric bootstrapping. Non-shading blocks indicate the absence of effector genes (**Table S1**). Clades with bootstrap support < 30% are collapsed into polytomies. Branch bubbles represent bootstrap support for the remaining clades (30%–100% support). Clustered groups were constructed from DAPCs. Non-*P. nodorum* isolates have their abbreviated species names in brackets. Isolates from France, USA and Denmark are indicated by black circles, triangles and a square, respectively. No group assignment was done for the isolates 16FG162 and 16FG170 as they were removed from the cluster analysis due to clonality.

An UPGMA tree was generated to examine the phylogenetic relationship between all fungal isolates (**Figure 2**). The *P. nodorum* isolates clustered away from the other species confirming the validity of the species. Amongst the *P. nodorum* isolates, most Australian *P. nodorum* isolates were connected by relatively long branches, indicating a high level of genetic diversification. Apart from two isolates (US isolate CP2052 and French isolate Fr_13_1) that clustered with the Australian, all the non-Australian isolates were located on distinct clades (**Figure 2**).

### Evidence of Core and Transient Populations in the Australian *P. nodorum* Panel

**Figure 3:**
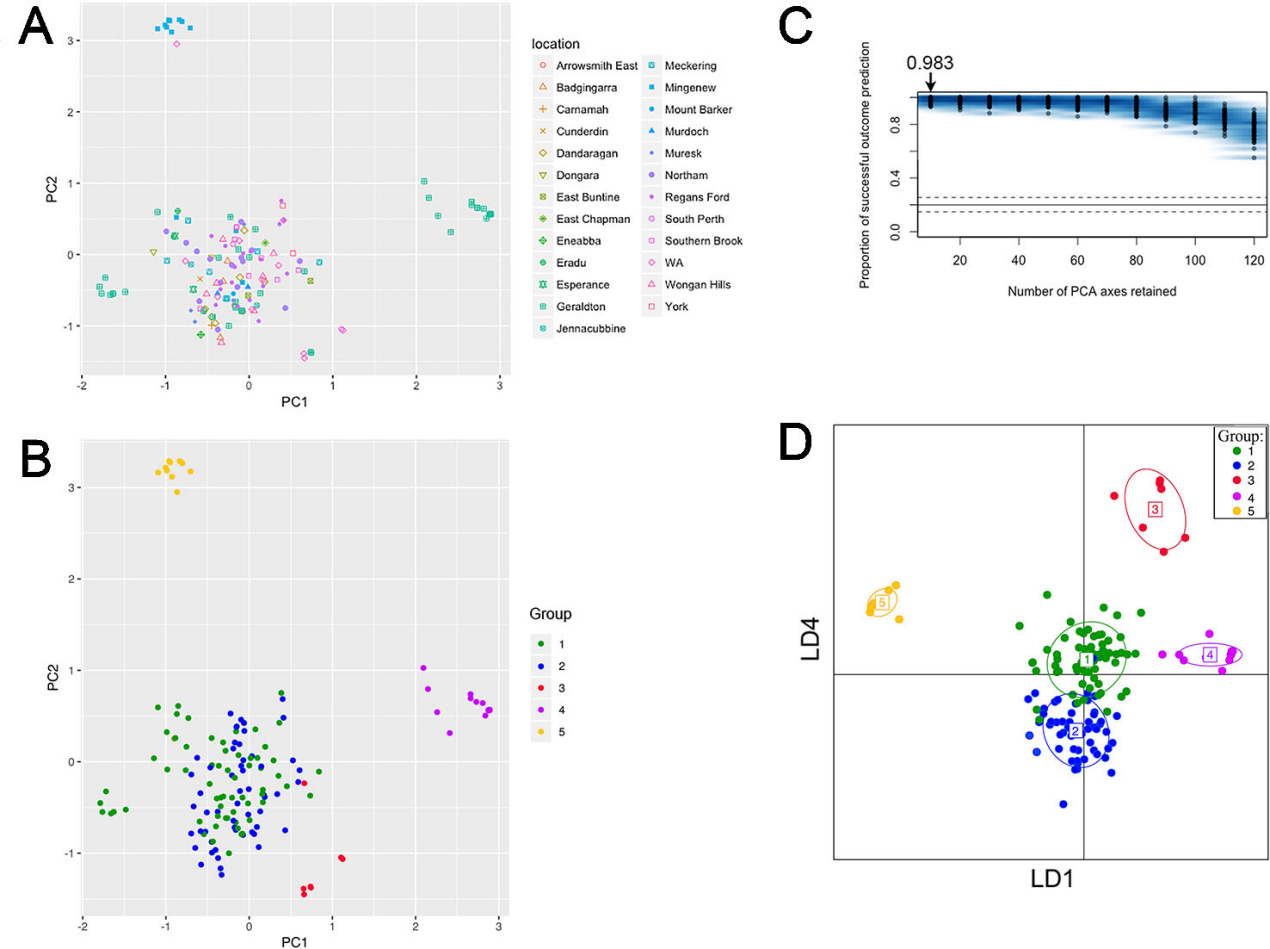
PC and DAPC analyses of population structure among 153 clone-corrected Australian *P. nodorum* isolates. First two PCs are shown where each point represents an isolate. **(A)** Sampling locations are indicated. **(B)** Isolates presented by genetic groups based on unbiased maximum-likelihood genetic clustering. **(C)** DAPC cross-validation test was performed for 10 to 120 PCs. Optimal number PCs to achieve the highest proportion of correct prediction outcome for the five-group model is shown. **(D)** PC1 to PC10 were DAPC-transformed to generate a simulated scatterplot displayed as LD1 and LD4 functions.

PCA revealed five groups were observed in the scatterplot built from PC1 (7.4% of the variance) and PC2 (6.6% of the variance) (**Figure 3A** and **Table S4**). The low levels of variance explained by PC1 and PC2 were possibly due to the large and complex diversity that exists in the collection, it is therefore necessary to use snapclust and supervised DAPC methods with multiple PCAs for further analysis. Snapclust analysis divided the population into five groups as presented in **Figure 3B**. The groups were further interrogated using DAPC. The DAPC models were evaluated by excluding isolates from training and predicting their group assignment (cross-validation). The level of correct reassignment was high for the selected five *P. nodorum* group model, with the maximum mean of successful prediction (0.983) obtained using the first 10 PCs only (**Figure 3C**). All subsequent DAPC analyses used the 10 PCs (explaining 39.3% of the total variance).

Comparative analysis between groups using DAPC indicated that Groups 1 and 2 were closely related, whereas Groups 3, 4, and 5 were distant from each other and from Groups 1 and 2 (**Figure 3D**). DAPC retained the five-group structure with only two isolates (15FG33 and WAC2285) reassigned between Group 1 (*n* = 56) and 2 (*n* = 66). Isolates assigned to Group 3 (*n* = 7), 4 (*n* = 14) and 5 (*n* = 10) using snapclust remained unchanged with DAPC.

**Figure 4:**
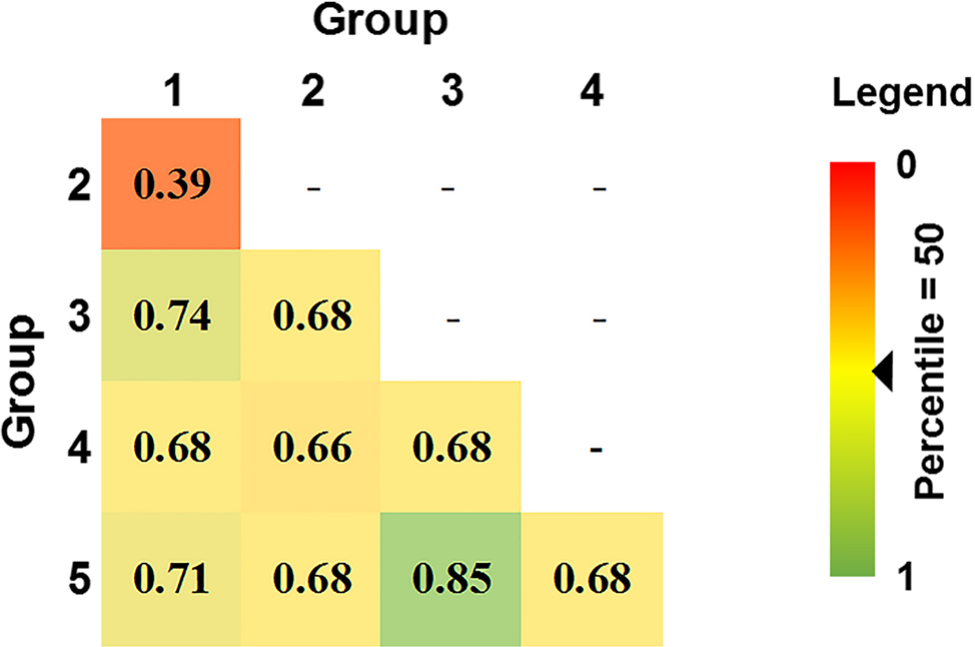
Pair-wise genetic distances between five DAPC Australian *P. nodorum* groups using Prevosti's distance. Genetic distances are ranked by percentile score. 0 and 1 denotes 0 and 100^th^ percentile, respectively.

80% of isolates were members of groups 1 and 2 and were embedded throughout the phylogenetic tree, while groups 3, 4, and 5 formed distinct clades (**Figure 2**). Overall genetic diversity within Groups 1 and 2 was high (mean H_exp_ = 0.69), but lower within Groups 3, 4, 5 (H_exp_ = 0.31, 0.20, and 0.09) (**Table 2**). Assessment of the genetic distance between *P. nodorum* groups using the Prevosti's model-free method indicates a higher level of genetic similarity between Groups 1 and 2 (0.39) compared with Groups 3, 4, 5 (0.66 to 0.85) (Prevosti et al., 1975) (**Figure 4**). The high level of genetic distances indicated limited gene flow between Groups 1/2 and Groups 3/4/5.

**Figure 5:**
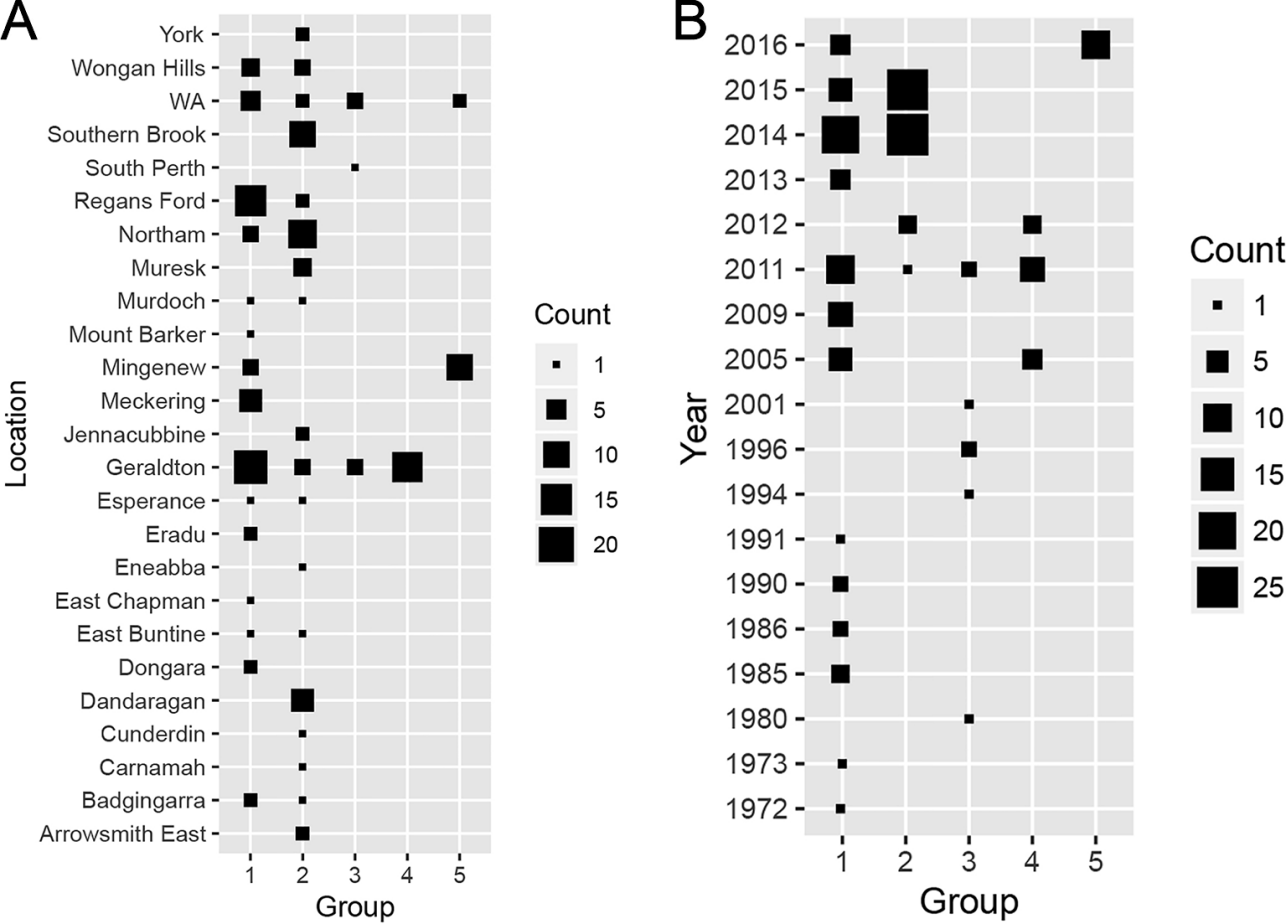
Distribution of discriminant analysis of principal components (DAPC)-grouped Australian *P. nodorum* isolates over sampling **(A)** locations and **(B)** times.

When the distributions of each group across sampling locations (**Figure 5A**) was considered, Groups 1 and 2 comprised isolates collected from most sampling locations. Group 3 consisted isolates sampled from two known locations (South Perth and Geraldton) and three isolates from unknown locations in WA. All Group 4 members derived from Geraldton whereas most Group 5 isolates were sampled from Mingenew. We then determined if the population structure was subjected to shifts over time (**Figure 5B**). Group 1 consisted of isolates sampled between 1972 and 2016. Group 2 consisted of isolates collected between 2011 and 2015. Members of Group 3 were found between 1994 and 2011. Isolates from Group 4 were sampled between 2005 and 2011. All isolates belonging to Group 5 were sampled in 2016. We concluded that Group 1 is ubiquitous in time and space and form the core Australian *P. nodorum* population whereas Groups 3 and 4 are transient and limited to specific time and locations. Groups 2 and 5 consisted of emerging isolates that became prominent since 2014 however, isolates belonging to the former group are much more prevalent from samples collected. Therefore, it can be concluded that Groups 1 and 2 form the core populations whereas Groups 3, 4, and 5 are considered smaller non-core populations. It can further be deduced that Groups 1 and 5 are emergent populations whereas Groups 3 and 4 are considered transient.

A DAPC analysis of the Australian isolate collection was also performed with non-Australian *P. nodorum* isolates using geographically defined groupings. It was observed that non-Australian isolates are distinct from Australian isolates (**Figure S3**).

### Determination of Mating Type Distribution and I_A_

We used PCR to assign mating types to our panel. A χ^2^ test demonstrated that the distribution of *MAT1-1* and *MAT1-2* across Groups 1 and 2 fitted the 1:1 ratio (**Table 3**). In contrast, isolates from Groups 3, 4, 5 had only a single mating type; Groups 3 and 5 carried *MAT1-1* while Group 4 carried *MAT1-2* (**Table 3**).

**Table 3:** I_A,_ rbarD and mating type assignments of the Australian *P. nodorum* groups.

Population	N	I_A_	rbarD	*P* (rbarD)	*MAT1-1*	*MAT1-2*	*P* (χ2)
Group 1	66	0.83	0.03	0.001	32	33	0.90
Group 2	56	0.61	0.02	0.001	25	31	0.42
Group 3	7	1.72	0.10	0.004	7	0	0.01
Group 4	14	1.80	0.11	0.001	0	14	0.00
Group 5	10	0.07	0.01	0.371	10	0	0.00
Total	153	0.99	0.04	0.001	74	78	0.75

rbarD is the standardised I_A_ denoted as ȓ_d_ (**Figure S4**).

I_A_ was determined for all groups of *P. nodorum* and rbarD significance test were used to determine if a population is in linkage disequilibrium. For this test, *P* < 0.05 would reject the null hypothesis of no linkage among markers (**Figure S4**). Difference to the mating type ratio and the rbarD significant test revealed that all Australian *P. nodorum* groups except Group 5 were in linkage disequilibrium signifying that the assortative recombination level among these groups is not high (Goss et al., 2014). Finding from this analysis indicated that sexual reproduction possibly only happened in Group 5 whereas asexual sporulation likely to be the main mode of reproduction in the other four groups.

### Shifts in *P. nodorum* Population Structure Correspond to Wheat Cultivar Adoption

**Figure 6:**
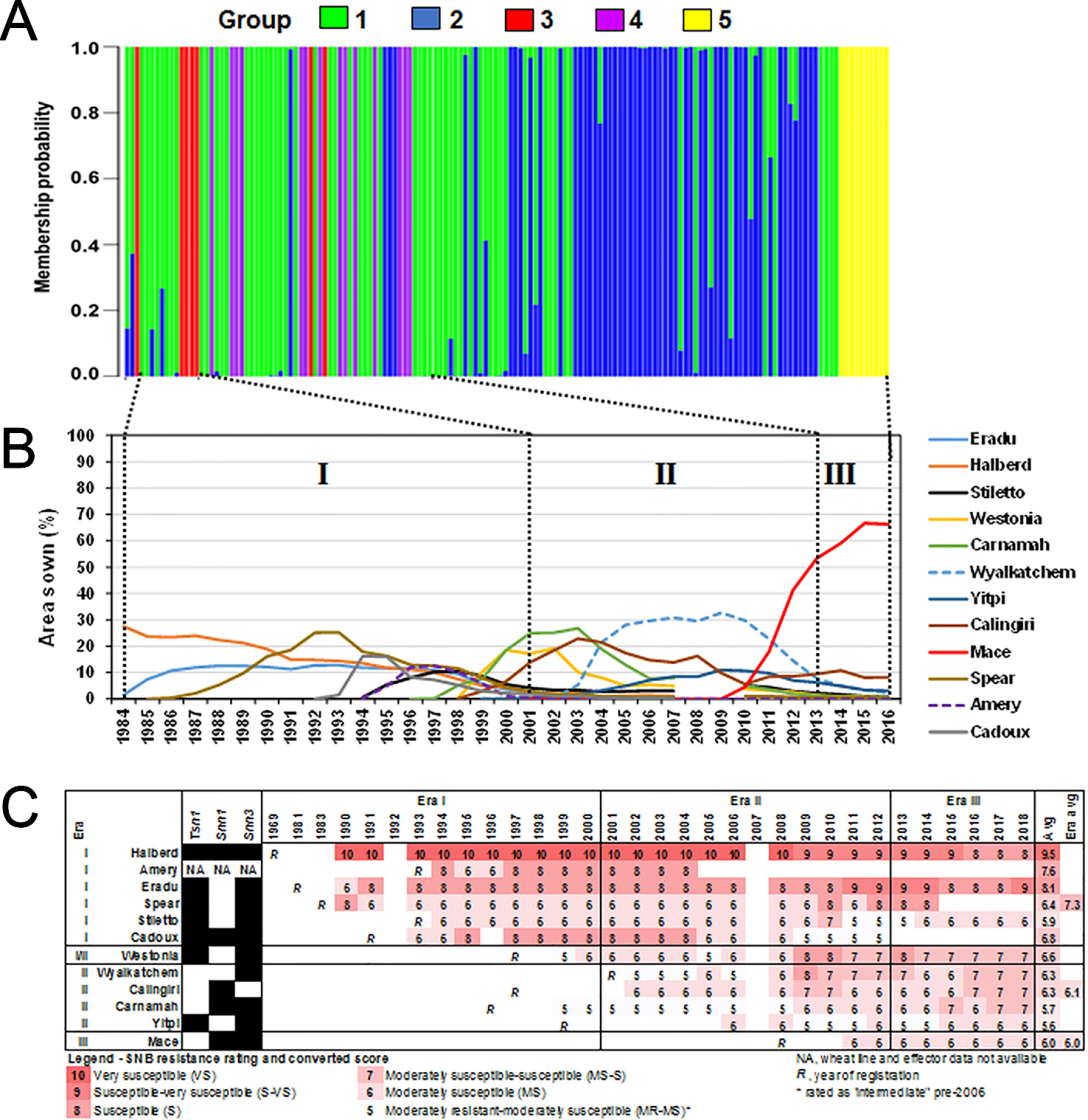
Shifts in the *P. nodorum* population structure over time using **(A)** discriminant analysis of principal components (DAPC) membership probability. Isolates were arranged in chronological order from 1976 (left) to 2016 (right). **(B)** Major wheats sown in WA between 1984–2016 (CBH Group, Australia). Wheats grown on at least 10% of the total area sown in any given year were shown. Roman numerals indicate designated eras of major changes in wheat cultivar adoption coinciding with shifts in the *P. nodorum* population. **(C)** SNB resistance rating of major wheats from 1990 to 2018 (DPIRD, Australia).

In an attempt to rationalise the *P. nodorum* population structure, we compared it to the prevalence of wheat cultivars where data was available (**Figures 6A, B**). Only wheat cultivars with ≥10% of the area sown in any one year were selected for analysis. We divided the period 1984 to date into three eras based on changes in cultivar adoption. Cultivars Eradu, Halberd, Calingiri dominated Era I. Carnamah, Spear, and Wyalkatchem defined Era II whereas Era III has a remarkable predominance of a single cultivar Mace. Groups 3 and 4 isolates were found in Eras I and II whereas Group 5 only emerged in Era III. A correlation coefficient test indicated significant associations between the emergence of Groups 3, 4, 5 and the frequency of wheat cultivar adoption in Era I, II and III (r = 0.89, df = 29, p-value = 3.01e^-11^ for wheats in Era I and Group 3; *r* = 0.83, df = 29, *P* = 9.22e^-9^ for wheats in Era II and Group 4; *r* = 0.83, df = 29, *P* = 9.15e^-9^ for wheats in Era III and Group 5).

We then examined the SNB resistance rating of popular wheat cultivars between 1990 and 2018 (**Figure 6C**) as determined by official DPIRD trials. Popular cultivars in Era I were on average at least one unit more susceptible to SNB than popular cultivars in Era II and Era III, indicating a slow but significant overall improvement in wheat breeding for SNB. In seven cases (Amery, Eradu, Cadoux, Westonia, Wyalkatchem, Calingiri, and Carnamah), the cultivar resistance rating declined in the years after release. The most dramatic cases are Eradu and Wyalkatchem which declined 3 units during the period of adoption. In three other cases (Halberd, Yitpi, and Mace) there was no sustained change during the period of use. There is some evidence of an increase in resistance rating after a cultivar had been dropped (Halberd and Cadoux).

### Whole Plant Infection Assay

**Figure 7:**
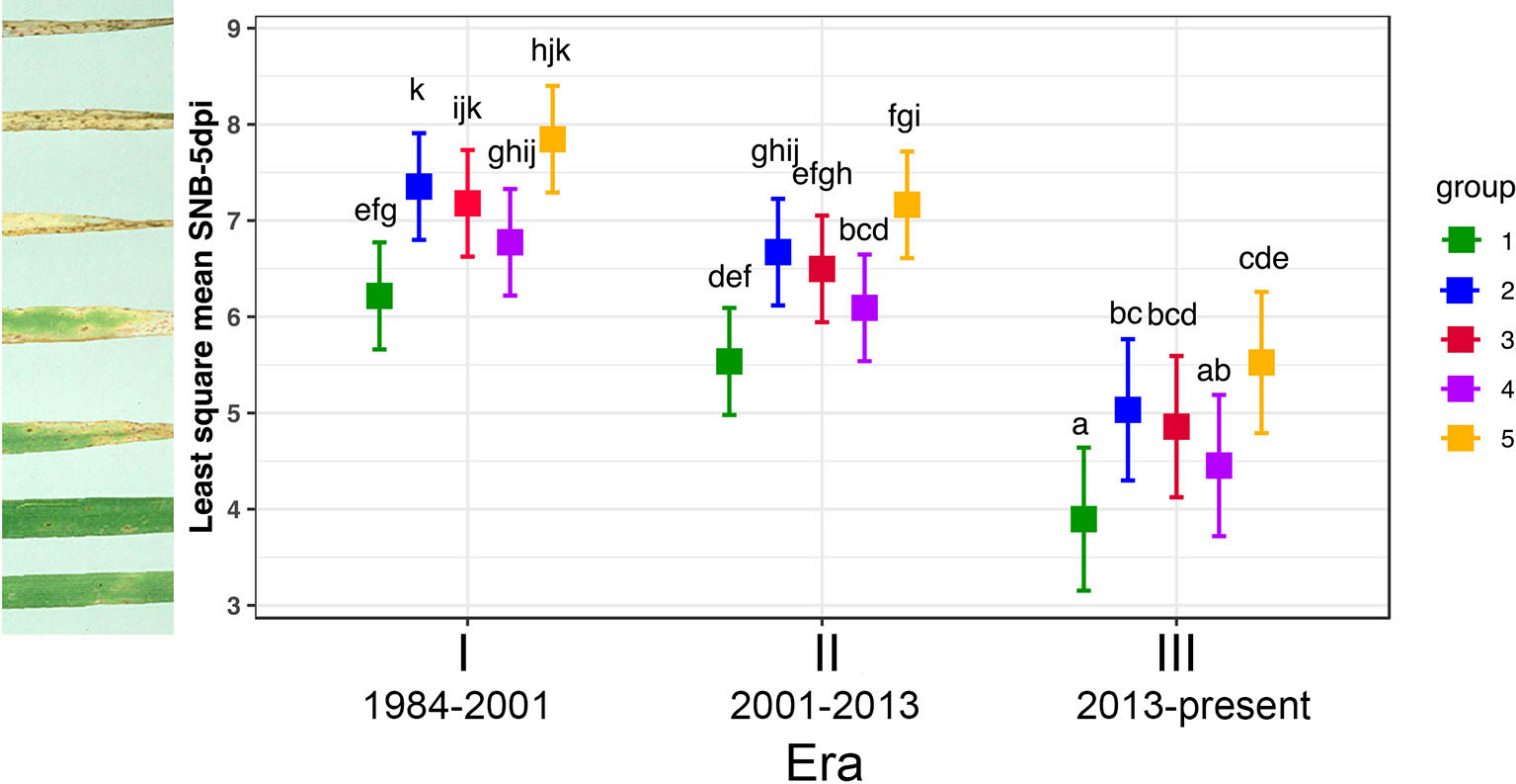
Tukey's Post Hoc tests on average virulence score of three *P. nodorum* isolates from each genotype group infecting seven representative wheat lines from three Eras. Levels not connected by the same letter are significantly different (*P* < 0.05). Individual disease scores are described in **Table S5**.

To identify factors that promote the formation of the three transient groups which coincided with the three Eras of mass adoption of a few or a single top-grown wheat lines from 1984 to 2016 and the shift of Group 1 to Group 2, we choose three isolates from each group to test their performance on the top-grown wheat lines during those periods. Data from the whole plant infection assay was split into two sets: disease scores of the three transient (Groups 3/4/5) and the core (Groups 1/2) on the same set of seven top-grown wheat lines (**Figure 7**). Of the seven wheat lines; Halberd, Eradu and Cadoux were the representatives for Era I, Carnamah, Calingiri and Wyalkatchem for Era II; and Mace for Era III.

Significant differences in isolates from different groups and wheat lines from the three Eras were observed when the disease data of Groups 1 and 2 was subjected to ANOVA, but there was no significant variation for their interactions (*P* = 1.13e^-04^, 2.12e^-04^, and 0.34, respectively). Since their interactions are not significant, two main factors: wheat lines from three Eras and isolates belonging to Groups 1 and 2 were analysed separately. Isolates from Group 1 were significantly less pathogenic than those from Group 2 (*P* = 2.12e^-04^, **Figure 7**) and wheat lines from Era III were significantly more resistant than those in Eras I and II (P = 1.13e^-04^, **Figure 7**).

For the three transient groups, ANOVA revealed that there are significant differences among isolates, wheat lines from different eras and their interactions (*P* = < 2e-16, 1.09e-07, and 8.93e-03, respectively). Since their interaction was significant, main effects (isolates and wheat lines from different Eras) were not considered independently and Tukey's Post Hoc test was deployed. **Figure 7** showed that all isolates from three eras were pathogenic and performed equally well on Era I wheat lines; for wheat lines in Era II, isolates from Groups 3 and 4 were similar (*P* = 0.99) and significantly less pathogenic than isolates from Group 5 (*P* = 0.04 and 0.006, respectively); in Era III, isolates from Group 5 were significantly more aggressive than those from Group 4 (*P* = 1.25e^-05^) and those from Group 4 were somewhere in the middle (**Figure 7**). Based on all wheat lines tested, Group 5 was the most aggressive with disease score significantly higher than all other groups, followed by the modern core Group 2 and the least pathogenic group was the old core Group 1 (*P* = 0.005).

## Discussion

SNB and tan spot are currently the two major fungal diseases of wheat in WA (Murray and Brennan, 2009). Both pathogens frequently co-exist on wheat and disease symptoms are often hard to distinguish (Loughman et al., 1993; Shankar et al., 2013). Difficulties in *P. nodorum* isolation prompted us to develop an effective method using boscalid to suppress *Ptr* to a sufficient level that allow *P. nodorum* growth from infected plant material to attain greater isolation efficiency.

In this study, we assembled a long time-span panel of isolates albeit the numbers of isolate from before 2000 is low. Such longitudinal collections are rare and we sought to determine how it could help us understand how the pathogen had evolved in the past few decades and assist in creating sound disease control strategies. Previous studies found that *P. nodorum* populations exist without any discernible structure even at the continental scales (Keller et al., 1997a; Keller et al., 1997b; Stukenbrock et al., 2006; Blixt et al., 2008). Keller et al. (1997a) examined the structure of field populations of *P. nodorum* from Texas, Oregon, and Switzerland and found 96% of the total genetic diversity within the geographically distant populations. Only 3% genetic dissimilarity was detected among nine wheat fields representing three geographical regions in Switzerland and different wheat cultivars (Keller et al., 1997b). We used robust statistical clustering methods to demonstrate that the Australian *P. nodorum* population distributed into five groups. To our knowledge, this is the first study to demonstrate evidence of a distinct genetic structure in a *P. nodorum* population.

Two isolates collected from Denmark and France were found to be closely related to Australian isolates (**Figure 2**). In a global *P. nodorum* population diversity study by Stukenbrock et al. (2006), it was reported that Australia is a sink population where traces of the pathogen from Europe were identified. As these two isolates clustered within Group 2 (**Figure 2**), it is possible that the ancestral lineage of these isolates were foreign incursions that contributed to the appearance and expansion of Group 2 isolates in the core population. More studies on European *P. nodorum* isolates are needed to verify this hypothesis.

Both mating types *MAT1-1* and *MAT1-2* were previously detected in the WA *P. nodorum* (Murphy et al., 2000; Solomon et al., 2004a) with a frequency not significantly different from 1:1, suggestive of regular sexual reproduction. Ascospores have been detected confirming that sexual reproduction does occur (Murphy et al., 2000; Bathgate and Loughman, 2001). This study also found an equal mixture of the two mating types in the entire collection but with a distinct pattern in the five groups. An equal ratio was found in both Groups 1 and 2. However, the I_A_ analysis rejected the null hypothesis of no linkage among markers tested, indicating that sexual reproduction is rare. This phenomenon could be due to Group 1 and 2 possess a high genetic diversity base with sexual reproduction commonly occurring in the past (Murphy et al., 2000, Bathgate and Loughman, 2001) and the diversity maintained over time with recently dominant asexual reproduction.

A strict pairing between isolates of similar genetic backgrounds is also another possible explanation for the observation. Numerous attempts to cross the isolates in our laboratory in different conditions reported to be suitable for the mating conditions of *P. nodorum* have been made without any success and differences in chromosome numbers are a common feature of plant fungal pathogens especially in these Dothideomycetes fungal species (Cooley and Caten, 1991; Galazka and Freitag, 2014; Bertazzoni et al., 2018; Moller et al., 2018). The possible restricted mating between similar isolates may have led to retention of equal mating type ratio and the low observed recombination frequency among different genetic backgrounds. If this is the case, Groups 1 and 2 pose the highest potential for rapid adaptation as we note that these groups possess greater genetic diversity and variants pools of known and potential effectors and virulence profiles.

Group 2 isolates were consistently more virulent than Group 1 and this effect has become more pronounced in Era III when cv. Mace has predominated. We suggest that the shift from Group 1 genotypes to the more contemporary Group 2 may be *via* selection for virulence on Mace (**Figure 6B**).

Groups 3, 4, and 5 each possessed a single mating type and a single ToxA, 1 and 3 isoform (**Table S1**). The small number of detected isolates compared to the core isolates, differences in virulence, low genetic variance within groups and the large distance between these groups may be explained by: changes in cultivar usage, population genetic bottlenecks, or new populations founded by a small number of well-adapted or foreign introduced isolates.

The origins of the non-core groups are currently being addressed. There are possibilities including they could be local diversifications since large genetic diversity also exists within the core groups which were adapted to the newly grown wheat lines; or they were derived from migration events from distant populations and selected due to the ability to infect a particular sets of wheat lines; or they were hybrids between local core and non-Australian isolates. Examining unique alleles for each of the five groups we found that the transient groups possess 14 unique and shared 135 common alleles with the two core Group 1 and 2 (**Table S6**). Although the numbers of isolates for Groups 3/4/5 are small, this study does indicate that among the available local and non-Australian candidates whoever is/are the most suitable for/adaptive to the current hosts at a particular time will prevail and expand and become a group of its own which is detectable.

The fact that Group 5 was collected only in 2016 and in one location, it is not absolutely certain that it is a transient based on time frame as data for post 2016 would be required. However, based on Group 5's properties of small number of isolates, limited spatial abundance, single mating-type and single ToxA, 1 and 3 isoform profile (**Table S1**), Group 5 seems to fit well with an emergent set. Likewise, Group 3 spanned over 30-year period but was considered transient due to its other characters such as small number of isolates, limited spatial abundance, single mating-type and single effector isoform profile. Additional data collected for the following years would be needed to warrant a more comprehensive understanding and more precise conclusions on Australian *P. nodorum* evolutionary processes and population genetic structure.

Shifts in fungal populations and disease expression on crops can be attributed to many factors including host shift/range expansion, foreign introductions and climatic changes (Elad and Pertot, 2014; Gladieux et al., 2015). The occurrence of SNB ‘boom-and-bust’ cycles in the last 44 years were evident when we combined evidence gathered from *P. nodorum* genotypes, wheat cultivar popularity and SNB resistance ratings. Released in 1969, Halberd remained a popular wheat cultivar until the late 90s and was highly susceptible to SNB and later replaced by Wyalkatchem. Reduction in the commercial adoption of Halberd resulted in a significant increase in the SNB resistance. We suspect the replacement of Halberd caused a shift in the *P. nodorum* population available at that time as it adapted. This pattern of the temporary appearance of the transient populations suggests that these groups were selected by virtue of their greater virulence on contemporary cultivars. The selection might explain the frequently observed decline in the resistance rating of the cultivars in the years following their peak adoption. The transient *P. nodorum* groups can reproduce asexually many times within a growing season which can result in an epidemic caused by a small subset of the population may be enough to skew the mating type ratio to the point where one mating type allele was dominant. It has been shown that the mating type locus is linked to virulence in other fungal pathogens (Lee et al., 1999; Hsueh and Heitman, 2008). The low genetic diversity and maybe absence of the other mating type would then render the transient populations vulnerable to the introduction of a new cultivar as it would be unable to generate the appropriate recombinants quickly enough. Instead, the diverse genetic pool of Groups 1 and 2 and/or new introductions from overseas may be able to compensate for their relative lack of virulence by their ability to recombine modestly virulent genotypes which leads to new and distinct transient groups capable to adapt to newly introduced cultivars.

SNB ratings of commercial wheat cultivars were scored on a ten-point scale (10 being “very susceptible”). Throughout the period in question, the highest resistance scores were 5, described as MR-MS. No cultivars have been assigned any better scores. Within that 5-point range, some cultivars declined in resistance by 3 units but 1 or 2 units was more common. We therefore describe this pattern of ubiquitous disease and modest declines in resistance over 5–10 years as a low amplitude boom and bust cycle in contrast to the classical full range boom and bust cycles seen for biotrophic diseases such as the cereal rusts (Agrios, 2005). As Brown (2015) points out, the classic boom and bust cycle is only found when single major R-genes are used to control a pathogen and where the loss of avirulence to the R-gene does not impact overall pathogenicity of the strain. Durable resistance has been achieved to biotrophic diseases like wheat powdery mildew and to SNB in the United Kingdom by the use of diverse germplasm sources and pragmatic selection for minor resistance genes based on field phenotyping. A similar breeding strategy was used for SNB in WA and was broadly successful in increasing the resistance levels and durability of adopted cultivars. Individual cultivars retained their resistance rating for 2–5 years and the reduction in the overall resistance rating was quite modest. The advent of effector-assisted breeding has accelerated the removal of susceptibility alleles and allowed more rapid breeding of cultivars with improved resistance to SNB and tan spot caused by *Ptr* through the removal of *Tsn1* (Vleeshouwers and Oliver, 2014).

This pattern of both non-core populations of specific and limited genetic diversity alongside with core populations of ubiquitous and rich in genetic variation implies that the selection of isolates used in the testing of new breeding lines and cultivars can be improved. The current system uses an uncontrolled mixture of stored and newer isolates (Shankar *pers. comm.*). Our studies indicate that the *P. nodorum* population from WA can be divided in to five groups. The transient groups from the past (Groups 3 and 4) can be safely ignored as they have been apparently driven to extinction. The first priority would be to screen for resistance to the current dominant emergent Group 5. We can predict that the use of cultivar/s with good resistance to Group 5 will lead to its rapid elimination and its replacement from within the core populations and/or foreign and/or local/foreign hybrids. Hence to achieve long term and durable resistance new cultivars should also be selected for resistance to Groups 1 and 2.

In more general terms, these studies indicate that the annual collection of isolates should be a priority for the control of all crop diseases. Examination of neutral genetic markers can be used to estimate population differentiation. Detection of skewed mating type ratios within sub-groups is a predictor of rapid adaptation to a current cultivar or other agronomic factor, such as a fungicide regime. Overall though these studies emphasise the value of cultivar diversity even in the absence of high amplitude boom and bust cycles (Wolfe, 1985; van den Bosch et al., 2014).

## Data Availability Statement

The datasets generated for this study can be found as supplemental data.

## Author Contributions

K-CT and HP conceived the experiment. K-CT, HP, and RO wrote the manuscript. HP, DJ, KR, and KD conducted all experiments. Results were analysed by K-CT, HP, PB, RO, and DJ. FL-R, M-HL, RV, PB, LG, and RO provided intellectual feedback and edited the manuscript.

## Funding

This study was supported by CCDM, a joint initiative of Curtin University and the Grains Research and Development Corporation (CUR00023).

## Conflict of Interest

The authors declare that the research was conducted in the absence of any commercial or financial relationships that could be construed as a potential conflict of interest.
