# Supplementary material for: Low Amplitude Boom-and-Bust Cycles Define the Septoria Nodorum Blotch Interaction
Source: Front Plant Sci. 2020 Jan 31;10:1785. doi: 10.3389/fpls.2019.01785 (PMC7005668; doi:10.3389/fpls.2019.01785)
Supplement: Supplementary file 1 [file Image_1.pdf]

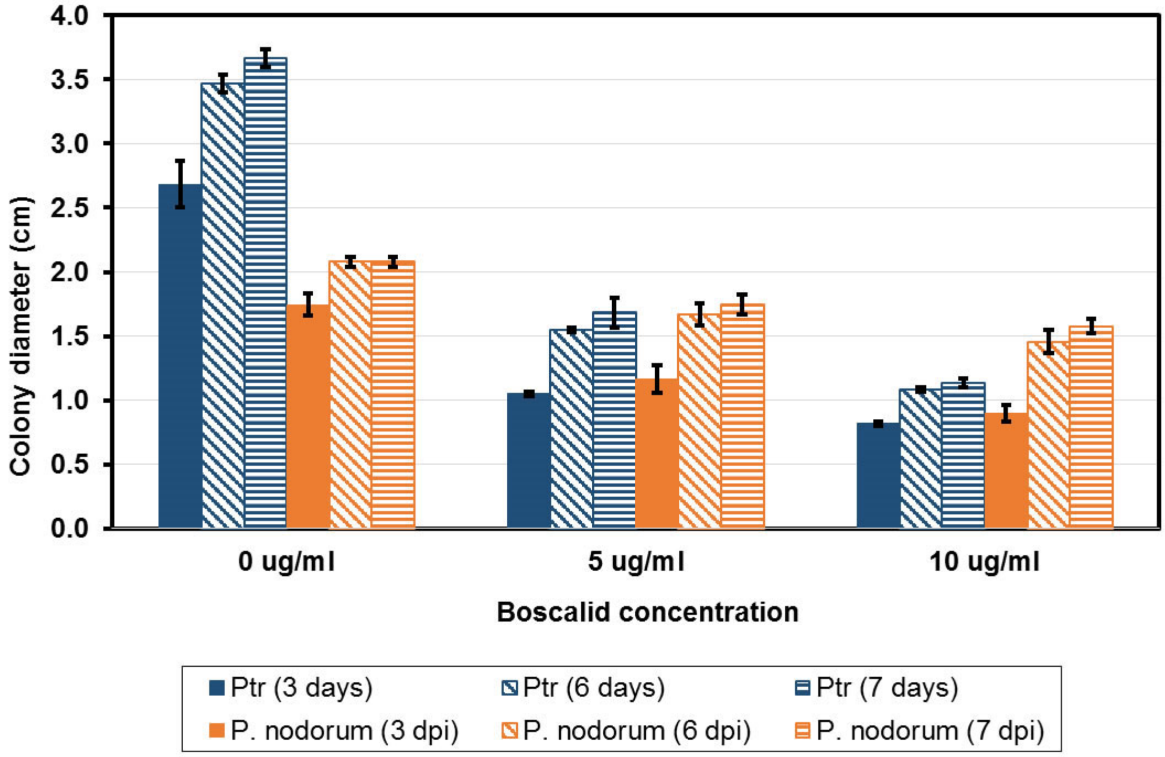

Figure S1. Radial growth measurements of *P. nodorum* and *Ptr* inoculated onto tap water agar with and without the fungicide boscalid.
