# Supplementary material for: Low Amplitude Boom-and-Bust Cycles Define the Septoria Nodorum Blotch Interaction
Source: Front Plant Sci. 2020 Jan 31;10:1785. doi: 10.3389/fpls.2019.01785 (PMC7005668; doi:10.3389/fpls.2019.01785)
Supplement: Supplementary file 2 [file Image_2.pdf]

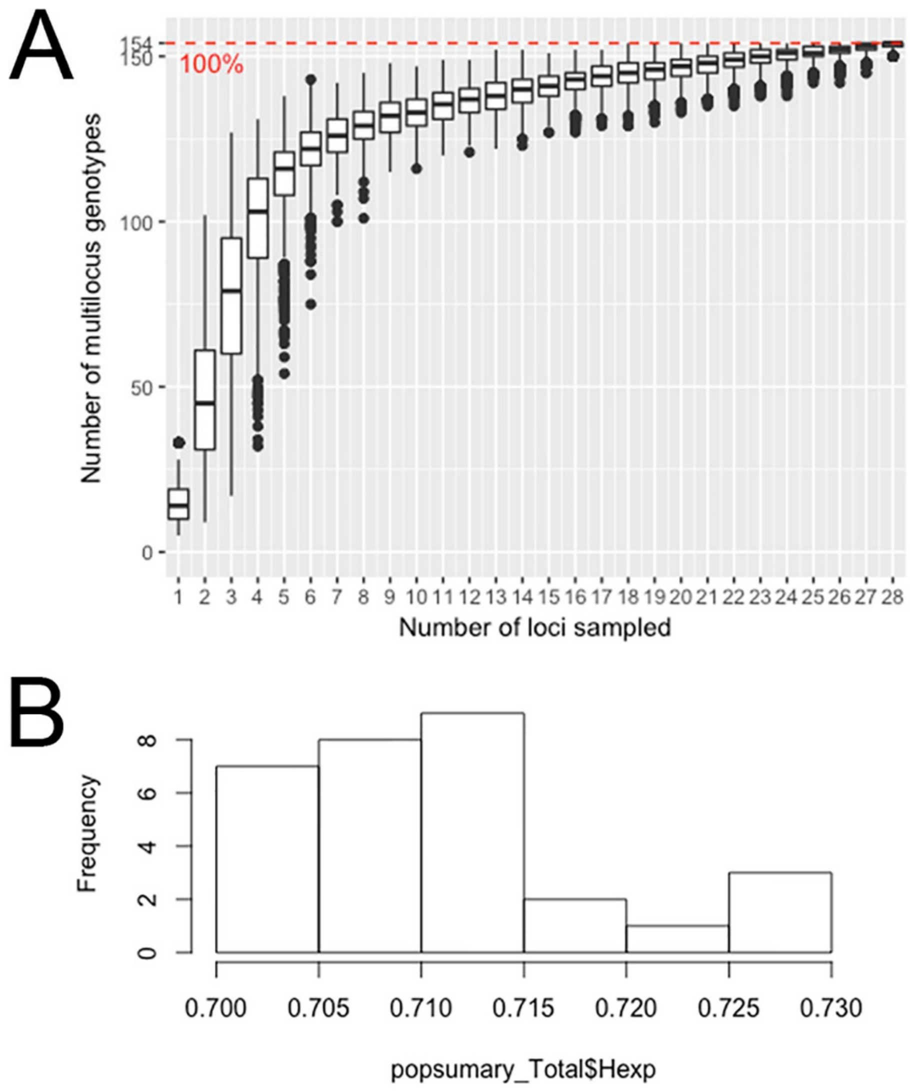

Figure S2. Informative accumulation of SSR markers. A. Genotype accumulation curve showing the percentage of discrimination ability between individuals of *n* loci. B. Nei's unbiased gene diversity produced by sequentially subtracting each SSR marker for 28 analysed markers.
