# Supplementary material for: Low Amplitude Boom-and-Bust Cycles Define the Septoria Nodorum Blotch Interaction
Source: Front Plant Sci. 2020 Jan 31;10:1785. doi: 10.3389/fpls.2019.01785 (PMC7005668; doi:10.3389/fpls.2019.01785)
Supplement: Supplementary file 3 [file Image_3.pdf]

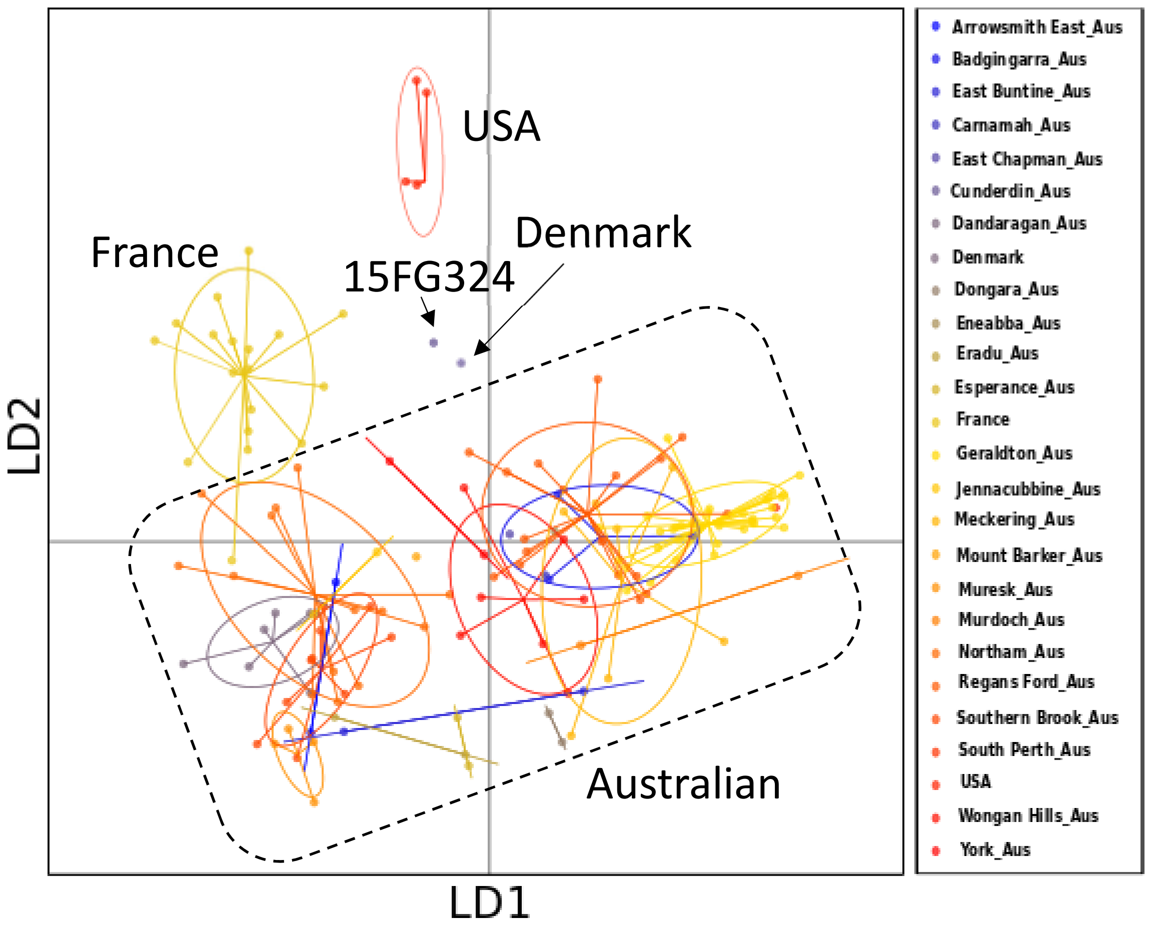

Figure S3. DAPC location-based analyses of population structure among 153 clone-corrected Australian *P. nodorum* isolates; and eighteen, four and one isolates from France, USA and Denmark, respectively. PC1 to PC10 were DAPC-transformed to generate a simulated scatterplot displayed as LD1 and LD2 functions.
