# Supplementary material for: Low Amplitude Boom-and-Bust Cycles Define the Septoria Nodorum Blotch Interaction
Source: Front Plant Sci. 2020 Jan 31;10:1785. doi: 10.3389/fpls.2019.01785 (PMC7005668; doi:10.3389/fpls.2019.01785)
Supplement: Supplementary file 4 [file Image_4.pdf]

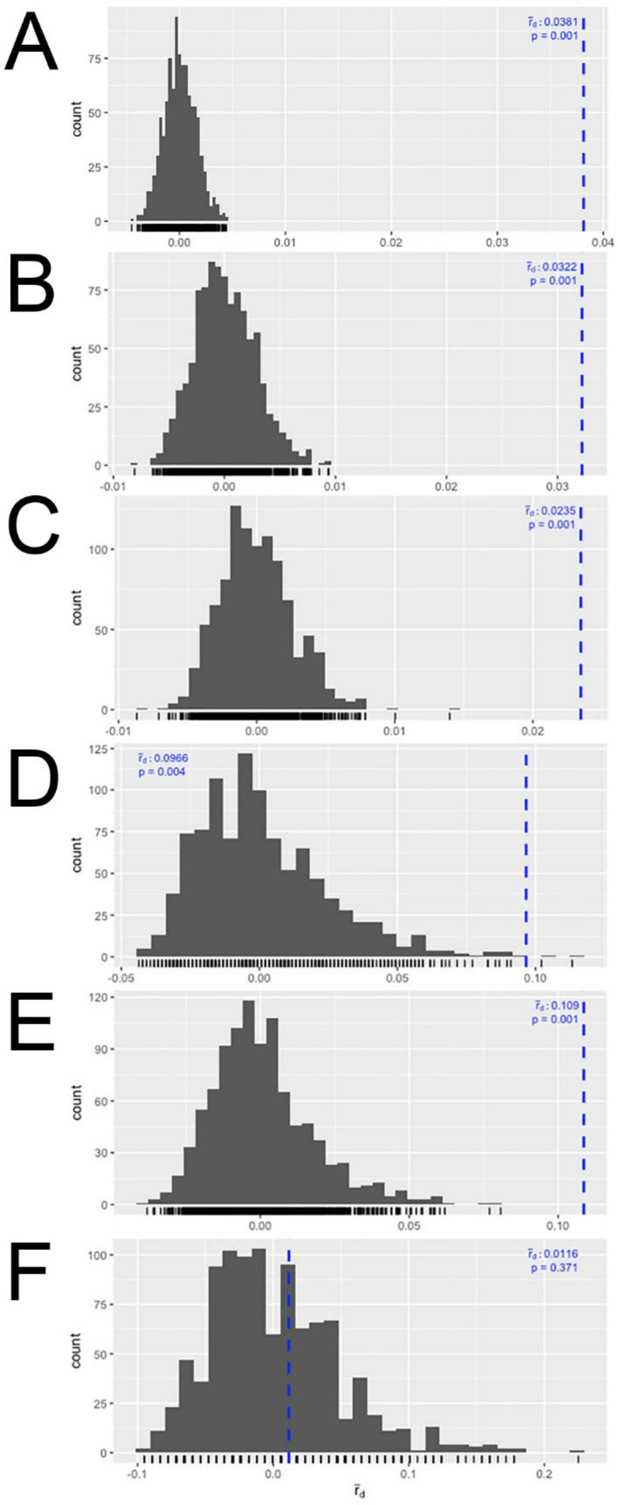

Figure S4. I_A testing of *P. nodorum* populations. I_A plots were constructed for (A) all non-clonal Australian *P. nodorum* isolates (B) Group 1, (C) Group 2, (D) Group 3, (E) Group 4 and (F) Group 5. *P* value and $\hat{r}_d$ generated using 999 permutations of the data are indicated in blue.
