# Supplementary material for: Low Amplitude Boom-and-Bust Cycles Define the Septoria Nodorum Blotch Interaction
Source: Front Plant Sci. 2020 Jan 31;10:1785. doi: 10.3389/fpls.2019.01785 (PMC7005668; doi:10.3389/fpls.2019.01785)
Supplement: Supplementary file 7 [file Table_3.docx]

**Table S3.** EC_50_ value of boscalid on *P. nodorum* isolates isolated with and without boscalid. When concentration of boscalid that inhibits 50% of fungal growth (EC_50_) was determined, no-significant differences in the EC_50_ of isolates that were obtained with and without boscalid.

| **Year** | **Location** | **Isolate** | **EC_50_ µg ml^-1^ boscalid** | **Isolation using boscalid?** |
| --- | --- | --- | --- | --- |
| 2001 | WA | SN15 | 2.26 | No |
| 2005 | Geraldton | WAC13070 | 2.58 | No |
| 2009 | Meckering | Meck1 | 2.31 | No |
| 2013 | Dongara | WAC13690 | 2.41 | No |
| 2014 | East Buntine | 53FG143 | 1.95 | No |
| 2015 | Muresk | 15FG47 | 2.22 | No |
| 2015 | Northam | 15FG031 | 2.22 | No |
| 2014 | Dandaragan | 15FG101 | 1.92 | Yes |
| 2015 | Dandaragan | 15FG100 | 2.28 | Yes |
| 2015 | Eradu | 15FG229 | 2.3 | Yes |
| 2015 | Esperance | 15FG237 | 2.26 | Yes |
